# Comparison of single nucleotide polymorphisms in the 3′ untranslated region of HLA-G in placentas between spontaneous preterm birth and preeclampsia

**DOI:** 10.1186/s13104-018-3280-2

**Published:** 2018-03-14

**Authors:** Ji Young Lee, Hyun Mi Kim, Mi Ju Kim, Hyun-Hwa Cha, Won Joon Seong

**Affiliations:** Department of Obstetrics and Gynecology, Kyungpook National University Hospital, Kyungpook National University School of Medicine, 130 Dongdeok-ro, Jung-gu, Daegu, 700-721 South Korea

**Keywords:** HLA-G, Single nucleotide polymorphism, Placenta, Preeclampsia, Preterm births

## Abstract

**Objective:**

To compare single nucleotide polymorphisms (SNPs) in the 3′-untranslated region (3′UTR) of human leukocyte antigen (HLA)-G in placentas between spontaneous preterm birth and preeclampsia pregnancies.

**Results:**

Placental samples matched for gestational age were obtained from 20 cases of spontaneous preterm births and 19 cases of preeclampsia. Genomic deoxyribonucleic acid was extracted from placenta tissue and the 3′UTR region of HLA-G was amplified via polymerase chain reaction. Nine SNPs were analyzed by direct Sanger sequencing. There was no significant difference in gestational age at delivery or birth weight between two groups. And there were no significant differences in the allele and phenotype frequencies between two groups.

## Introduction

Preterm births and preeclampsia (PE) are the major complications that contribute to morbidities during pregnancy; however, its pathophysiology has not been clearly identified. Immunological maladaptation in the maternal–fetal interface has accounted for the pathogenesis of adverse pregnancy outcomes, including PE, intrauterine growth restrictions, spontaneous preterm births, and congenital infections [1]. The human leukocyte antigen (HLA) system is a major histocompatibility complex (MHC) protein in humans. Among the HLA antigens, the human leukocyte antigen-G (HLA-G) antigen is expressed only in humans and has a proposed role in protecting the extravillous trophoblast from the maternal immune system [2, 3]. In addition, several researchers have reported that reduced expression of HLA-G is linked to PE [4–8], and the HLA-G protein has been reported to be linked to spontaneous preterm birth and intra-amniotic inflammation or infections [9, 10]. Single nucleotide polymorphisms (SNPs) are the most common type of genetic variations and are defined by at least two variants, one of which is present at a frequency greater than 1% [11, 12]. Recently, associations between preeclampsia and SNPs in the 3′ untranslated region (3′UTR) of HLA-G have been observed [13]. However, these studies only compared placentas from cases of preeclampsia and normal full term pregnancies without consideration of gestational age at delivery. Therefore, the aim of the present study was to compare SNPs in the 3′UTR of the HLA-G between pregnancies complicated with spontaneous preterm births and those with preeclampsia in gestational age matched placentas.

## Main text

### Methods

#### Tissue collection

We collected placental samples from 20 cases with spontaneous preterm births and 19 cases of preeclampsia. The placental samples 1 cm × 1 cm in size were aseptically obtained from the fetal side and kept in a frozen state (− 70 °C). In all cases of preeclampsia in this study, the patients had blood pressures ≥ 140/90 mmHg, dipstick protein ≥ 1+/4+, or 24-h urine protein ≥ 300 mg/dl. For gestational age matching, cases of spontaneous preterm birth were selected with a similar gestational age at delivery for each case of preeclampsia. The indications for delivery in preterm birth group included preterm labor and preterm premature rupture of the membranes without other maternal or fetal indications. All pregnant women enrolled in this study gave written informed consent before participation. This study was approved by the Institutional Review Board (IRB) of Kyungpook National University, Daegu, South Korea (IRB File No: 2016-07-005).

#### Deoxyribo nucleic acid (DNA) extraction

Genomic DNA was extracted from placenta tissue, using the Qiagen QIAamp Fast DNA tissue Kit (Qiagen, Valencia, CA, USA). DNA concentration was determined using a NanoDrop ND-1000 spectrophotometer(Thermo Fisher Scientific, Wilmington, DE) and purity was assessed based on the 260/280 nm absorbance ratio from 1.7 to 2.1.

#### Polymerase chain reaction (PCR) and PCR product purification

Nine SNPs in the 3′UTR of HLA-G that were previously described [13] were evaluated in this study as follows; rs371194629, rs1707, rs1710, rs17179101, rs17179108, rs1063320, rs9380142, rs1610696, rs1233331. The DNA sequences including the 3′UTR region of HLA-G were amplified via PCR in a final reaction volume of 50 μl, using the Multiplex PCR Master Mix (Qiagen, Valencia, CA, USA) and 10 pmol of each primeralong with 100 ng of genomic DNA using SimpliAmp™ Thermal Cycler(Life Technologies, Carlsbad, CA, USA). Size of PCR amplicon was 526 bp. The PCR parameters were as follows: initial denaturation of 95 °C for 15 min, followed by 35 cycles of 94 °C for 30 s, 60 °C for 90 s, 72 °C for 40 s, and a final extension of 72 °C for 10 min. Amplified PCR products were purified using the GeneAll expin Kit (GeneALL Biotechnology, Seoul, Korea.).

#### Genotyping by Sanger sequencing

Sanger sequencing was performed using the BigDye Terminator V 3.1 Cycle Sequencing Kit (Applied Biosystems, USA). Sequencing results were compared with reference sequences (HLA-G/NM_002127/ENSG00000204632/ENST00000428701.5), using the alignment program BLAST V2.5.0 of NCBI. The positions of nine SNP were manually compared using CodonCode Aligner V.5.1.5(CodonCode Corporation, Centerville, MA, USA).

#### Statistical analysis

The Hardy–Weinberg (HW) equilibrium and Chi square test were used to verify the significance of differences in the genotypic data and allelic frequencies between two groups. Chi square test was two-sided test and statistical significance was defined when p value was lower than 0.05. The Haploview software (V 4.2) was employed to perform the linkage disequilibrium (LD) and haplotype analysis among nine of the genotyped SNPs. LD patterns were constructed using an algorithm designed by Gabriel et al. [14] with a minor allel frequency (MAF) of ≥ 1%.

### Results

The clinical characteristics of women with spontaneous preterm subjects and PE are listed in Table 1. There were no significant differences between the two groups in terms of gestational age at delivery and birth weight (32.8 ± 4.8 vs. 33.3 ± 4.4, *p *= 0.74, 2.19 ± 0.9 vs. 1.80 ± 0.8, *p *= 0.17). Haploview showed the genotype distributions were in HW equilibrium except rs17179101 in pregnancies without preeclampsia. The allele frequencies of all the SNPs did not show any statistical differences between the two groups (Table 2). Furthermore, there were no statistical differences between the control group and cases of PE in terms of genotype frequencies for all nine SNPs (Table 3).

**Table 1:** Clinical characteristics of the subjects included in this study

	Spontaneous preterm birth (n = 20)	PE (n = 19)	*p* value
Age^a^ (year)	32.9 ± 3.8	33.7 ± 4.4	0.53
GAD^a^ (day)	32.8 ± 4.8	33.3 ± 4.4	0.74
Nulliparity (%)	11 (57.9)	6 (30.0)	0.08
Birth Weight^a^ (kg)	2.19 ± 0.9	1.80 ± 0.8	0.17

T test and Chi square test, both two-sided, was used to analyze the data and statistical significance was defined when p value was lower than 0.05

*PE* preeclampsia

^a^Mean ± standard deviation; GAD, gestational age at delivery

**Table 2:** Allele frequencies of the polymorphisms studied

	Spontaneous preterm birth		PE		p value
	n	%	n	%	
rs371194629					
del	33	82.5	31	81.6	0.916
ATTTTGTTCATGCGT	7	17.5	7	18.4	
rs1707					
T	38	95.0	38	100	0.494
C	2	5.0	0	–	
rs1710					
C	24	60.0	26	68.4	0.438
G	16	40.0	12	31.6	
rs17179101					
C	37	92.5	36	94.7	1.000
A	3	7.5	2	5.3	
rs17179108					
C	35	87.5	36	94.7	0.432
T	5	12.5	2	5.3	
rs1063320					
G	24	60.0	27	71.1	0.305
C	16	40.0	11	28.9	
rs9380142					
A	24	65.0	27	71.1	0.567
G	16	35.0	11	28.9	
rs1610696					
C	38	95.0	33	86.8	0.257
G	2	5.0	5	13.2	
rs1233331					
G	40	100	40	100	–
A	0	–	0	–	

Chi square test, both two-sided, was used to analyze the data and statistical significance was defined when p value was lower than 0.05

*PE* preeclampsia

**Table 3:** Genotype frequencies of the polymorphisms studied

	Spontaneous preterm birth		PE		p value
	n	%	n	%	
rs371194629					
del/del	14	70	13	68.4	0.994
ATTTTGTTCATGCGT/ATT	1	5	1	5.3	
del/ATTTTGTTCATGCGT	5	25	5	26.3	
rs1707					
T/T	18	90	19	100	0.487
C/C	–	–	–	–	
T/C	2	10	–	–	
rs1710					
C/C	9	45	9	47.4	0.461
G/G	5	25	2	10.5	
C/G	6	30	18	42.1	
rs17179101					
C/C	18	90	18	94.7	0.614
A/A	1	5	–	–	
C/A	1	5	1	5.3	
rs17179108					
C/C	16	80	18	94.7	0.547
T/T	3	15	–	–	
C/T	1	5	1	5.3	
rs1063320					
G/G	9	45	8	42.1	0.157
C/C	5	25	11	5.3	
G/C	6	30	0	52.6	
rs9380142					
A/A	10	50	9	47.4	0.297
G/G	4	20	1	5.3	
A/G	6	30	9	47.4	
rs1610696					
C/C	18	90	15	78.9	0.485
G/G	–	–	1	5.3	
C/G	2	10	3	15.8	
rs1233331					
G/G	20	100	19	100	–
A/A	–	–	–	–	

Chi square test, both two-sided, was used to analyze the data and statistical significance was defined when p value was lower than 0.05

*PE* preeclampsia

### Discussion

In this study, we compared nine SNPs in the 3′UTR region of HLA-G between spontaneous preterm birth and preeclampsia in gestational age matched placentas. And we found that there were no differences in allelic frequencies and genotypic distributions between the two groups. HLA-G has been reported to contribute to maternal immune tolerance at the maternal–fetal interface through natural killer cell receptors by blocking their cytotoxic effect [1]. Previously, SNPs in the 3′UTR region of HLA-G have been evaluated in terms of preeclampsia pathophysiology, which showed diverse results [15–20]. Among the nine variations, no statistically significant differences have been shown in genotype distributions for rs1610696, rs1707, rs1710, rs17179101, rs17179108, and rs1233331 between the control subjects and cases of PE [16]. In contrast, the distribution of the A or G allele of rs9380142 and the C or G allele of rs1063320 was found to be different between the control subjects and cases of PE [16, 20]. In particular, rs1704 has been analyzed in several studies [15–19, 21], which showed the presence of a 14-bp insertion that was significantly higher in cases of PE [16, 19, 21]. Recently, Quach et al. suggested that a pair of alleles in the 3′UTR of HLA-G (rs17179101 C/C allele and rs938021 G/G) may play a role in the pathophysiology of PE [13].

Furthermore, HLA-G has been investigated with regards to preterm birth or intraamniotic inflammations/infections. Soluble HLA-G (sHLA-G) has been detected in both the maternal serum/plasma and amniotic fluid, and its concentrations are influenced by the gestational age and several maternal or fetal complications, including fetal gender, fetal neural tube defect, intraamniotic inflammations/infections, and preeclampsia [7, 9, 22–24]. Kusanovic et al. investigated sHLA-G in amniotic fluid and found that it was elevated in preterm parturition and associated with intraamniotic inflammation/infection [9]. Additionally, higher vaginal and maternal serum concentration of sHLA-G in cases of preterm premature rupture of membranes may be associated with local or systemic inflammation [24]. Interestingly, the concentration of maternally circulating sHLA-G are significantly lower in patients with PE even before its manifestation [7, 23, 24]. Likewise, it is well known that HLA-G is involved in the pathophysiology of spontaneous preterm labor and PE; however, studies on HLA-G in spontaneous preterm birth have mainly investigated sHLA-G only, not the 3′UTR in HLA-G. Therefore, our study was meaningful because we analyzed and compared SNPs in the 3′UTR of HLA-G between placentas with spontaneous preterm birth and those with preeclampsia. In addition, we matched the gestational age at delivery, and the results showed similar birth weights between the two groups. Previous studies only compared HLA-G in the placentas from cases of PE versus normal term pregnancy and PE.

In conclusion, we found that there were no significant differences in the SNPs in the 3′UTR of *HLA*-*G* between spontaneous preterm birth and PE. Further studies with a larger cohort are warranted.

## Limitations

The presented data is limited by sample size. For gestational age matching, we did not compare preeclampsia with normal pregnancy, but compared spontaneous preterm birth with preeclampsia due to gestational age matching.

## Abbreviations

HLA-G: human leukocyte antigen-G
SNP: single nucleotide polymorphisms
3′UTR: 3′-untranslated region
PCR: polymerase chain reaction
PE: preeclampsia
MHC: major histocompatibility complex
